# Prevalence and correlates of *Mycoplasma genitalium* infection among patients attending a sexually transmitted infection clinic in Guangdong, China: a cross-sectional study

**DOI:** 10.1186/s12879-021-06349-4

**Published:** 2021-07-05

**Authors:** Xiao-Hui Zhang, Pei-Zhen Zhao, Wu-Jian Ke, Liu-Yuan Wang, Lai Sze Tso, Zheng-Yu Chen, Yu-Ying Liao, Chun-Mei Liang, Hui-Ru Chen, Xu-Qi Ren, Jin-Mei Huang, Jason J. Ong, Fan Yang, Li-Gang Yang

**Affiliations:** 1 Dermatology Hospital, Southern Medical University, Guangdong Province, Guangzhou, China; 2 Guangdong Provincial Dermatology Hospital, Guangdong Provincial Center for Skin Diseases and STD Control, Guangzhou, China; 3 Department of Culture Studies and Oriental Languages, University of Oslo, Oslo, Norway; 4 Anthropology, Massachusetts Institute of Technology, Cambridge, MA USA; 5 Center for Health and Human Development Studies, Sun Yat-sen University, Guangzhou, Guangdong China; 6 Clinical Research Department, London School of Hygiene and Tropical Medicine, London, UK; 7 Central Clinical School, Monash University, Melbourne, Australia; 8 University of North Carolina, UNC Project-China, Guangzhou, China

**Keywords:** Prevalence, *Mycoplasma genitalium*, Risk factor

## Abstract

**Background:**

*Mycoplasma genitalium* (MG) causes urogenital tract infections and is associated with reproductive morbidity. Although MG has been reported across many regions and population groups, it is not yet routinely tested for in China. Our study contributes to current research by reporting the prevalence and correlates of MG infection in patients attending a sexually transmitted infection (STI) clinic in Guangdong from Jan 2017-May 2018.

**Methods:**

Urethral (from 489 men) and endo-cervical (from 189 women) samples, blood samples, and patient histories (via questionnaires) were collected. Doctors clinically diagnosed anogenital warts (GW) during the examination (*n* = 678). The presence of MG was evaluated using an in-house via polymerase chain reaction protocol. We also tested all participants for herpes simplex virus-2 (HSV-2), *Neisseria gonorrhoeae* (NG), *Chlamydia trachomatis* (CT), syphilis and HIV. Univariate and multivariate logistic regression were used to evaluate factors associated with MG.

**Results:**

MG was detected in 7.2% (49/678) of the patients (men, 7.4%; women, 6.9%). The MG positivity rate was 14.2% among symptomatic patients, and 5.6% for asymptomatic patients, respectively. Only 36.7% (18/49) Mg positive patients were symptomatic. Among the MG-infected patients, 10.2% were co-infected with CT, 6.1% with NG, 8.2% with HSV-2, 4.1% with syphilis and 22.4% with GW. Presentation with clinical symptoms was significantly associated with MG infection [OR = 2.52 (2.03–3.13)]. In our analysis, MG was not associated with other STIs.

**Conclusions:**

MG is a relatively common infection among individuals attending an STI clinic in Guangdong Province. Routine testing of symptomatic patients may be necessary, and more epidemiological studies are needed to provide evidence for future testing guidelines.

## Background

*Mycoplasma genitalium* (MG), first isolated from urethral specimens of two men with non-gonococcal urethritis in 1981 [1], is the smallest free-living and self-replicating sexually transmitted pathogen [2]. Since this initial discovery, MG has been identified as a cause of acute and persistent non-gonococcal urethritis (NGU) [3] and cervicitis and is associated with prostatitis [4], balanoposthitis [5], proctitis [6–8] pelvic inflammatory disease (PID) [9], adverse pregnancy outcomes [10], and infertility in both men and women [11, 12]. MG infection may also increase HIV susceptibility [13].

Some existing epidemiological studies in China suggest that MG is widespread in China. In the available studies of MG infection in men living in China, the positive rate was 3.4% as detected in first-void urine (FVU) and 5.4% as detected in the rectal samples of men who have sex with men (MSM) in Shenzhen [14], 15.3% in the rectal samples of MSM in Shenyang [15]. Among men living with HIV in Jiangsu Province, the positive rate ranged from 20.1 to 29.2% as detected in FVU [16–19]. The positive rate of MG was 10.2% in symptomatic patients with NGU and 13.9% in patients with asymptomatic NGU in Hong Kong [20, 21] and was 28.1% in men with STD-related complaints in Guangxi [22]. MG prevalence data for women are even more limited than those for men. However, one study reported a 13.2% prevalence in female sex workers living in Jiangsu [23].

In China, MG testing is not routinely performed in most sexually transmitted infection (STI) clinics, as there is no standard MG screening protocol to guide clinical practice, and commercial diagnostic kits were not available until 2019. National prevalence data for MG among routine STI patients are thus lacking. Therefore, we performed a cross-sectional study among patients attending a major urban STI clinic in Guangzhou, Guangdong Province, to assess the prevalence and correlates for MG infection.

## Methods

### Study design

From January 2017 until May 2018, we recruited patients attending our STI clinic in the Dermatology Hospital of Southern Medical University. Because our STI clinic is busy, we invited only patients who went to designated consulting rooms to participate in the study. Only first-time visitors were included in the study, these patients were symptomatic individuals seeking STI screening or asymptomatic individuals seeking STI screening due to high-risk sexual behaviours or having had contact with an infected individual. Patients aged less than 18 years or reporting any systemic antibiotic treatment in the previous 30 days were excluded. After the participants provided informed consent, their sociodemographic characteristics, sexual behaviour data and clinical findings were collected and documented as part of routine clinical procedure.

### Sample size

The primary outcome of this study was the prevalence of MG. A previous study reported that the prevalence of MG was 11.2% [20]; thus, we calculated two-sided confidence intervals for one proportion to estimate a sample size of 651 for this study in order to produce a two-sided 95% confidence interval with a width of 0.050.

### Pathogen detection

Urethral swab specimens and FVU at least 2 h after the last previous urination were collected from all men, including those who reported no symptoms. The urethral swab specimens were used for urethral leucocyte count and MG detection. The urine specimens were tested for *Neisseria gonorrhoeae* (NG) and *Chlamydia trachomatis* (CT). Endo-cervical swab specimens were collected from women for MG/NG/CT nucleic acid testing and leucocyte count using microscopy. Blood sera were tested for HIV, *Treponema pallidum* (TP) and *herpes simplex virus* 2 (HSV-2) antibodies.

The amplification assay for MG detection was performed as follows: DNA was extracted using a commercial kit according to the manufacturer’s instructions (Daan, Guangzhou, China). MG was detected via TaqMan MGB real-time polymerase chain reaction (PCR) with a sensitivity of five genome equivalents (geq)/reaction, as described by Jensen et al. [24]. Tests for NG and CT were performed using Roche’s Cobas 4800 CT/NG test (Roche Diagnostics, Mannheim, Germany). Serum was tested for HIV using two antibody tests—a rapid HIV antibody test (Wantai, Beijing, China) and a second antibody test (Abon Biopharm, Hangzhou, China). If both were positive, another blood sample was collected and sent to the Guangzhou Center for Disease Control and Prevention for confirmation by Western blot analysis (MP Biomedical, Singapore). Serum was tested for syphilis using a toluidine red unheated serum test (TRUST, Rongsheng Bio-technology Limited Corporation, Shanghai, China) and *Treponema pallidum* particle agglutination (TPPA, Fujirebio Inc., Japan) test. Serum was tested for HSV-2 using the enzyme-linked immunosorbent assay (ELISA) method (Trinity Biotech, Guangzhou, China). Microscopic examination was performed on-site, and other samples were stored at − 20 °C before testing.

### Measures

Patients were considered symptomatic if they self-reported symptoms, such as dysuria, urethral pruritus,vaginal discharge, vaginal bleeding after sexual contact, and/or had abnormal findings during physical examination, such as urethral discharge (for males), abnormal vaginal discharge r yellowish mucopurulent t discharge at the cervical os and/or contact bleeding of the cervix (for females).

The urethral smear was regarded as presence of excess leucocytes when ≥5 polymorpho- nuclear leucocytes (PMNL) were seen per high power field (HPF) (× 1000) in ≥5 HPF, and the cervical smear was regarded as presence of excess leucocytes when > 30 PMNL were seen per HPF. Patients with positive TRUST and TPPA tests were considered to have an active syphilis infection unless there was a documented history of previously treated syphilis. Patients with positive HIV screening and confirmatory tests were considered to have HIV infection. Anogenital warts (GW) and anogenital herpes (GH) were diagnosed according to the diagnostic criteria for China’s “National Notifiable Disease Reporting System” (Version 2008). These diagnoses were based mainly on exposure history and consistent clinical findings.

### Ethics approval

The study was approved by the Ethics Committee of the Dermatology Hospital of Southern Medical University (approval no. GDDHLS-201502, 31/03/2015). The whole study including all its methods were carried out in accordance with relevant guidelines and regulations at the Dermatology Hospital of Southern Medical University, which are all in agreement with the Declaration of Helsinki. All data was anonymised prior to the analysis. Written informed consent was waived because the risk associated with participating in this study was deemed minimal and involved no procedures requiring consent outside of the context of participating in the study. Oral informed consent was obtained from all study participants prior to inclusion in the study. It is also in accordance with DHHS (45 CFR 46.117).

### Statistical analysis

All data were entered into EpiData 3.0 via double entry for statistical analysis. Descriptive analyses were conducted on the patients’ demographic characteristics and the prevalence of MG and other STIs. Categorical variables are presented as numerical and percentage values. Continuous variables are expressed as $\overline{x}\pm SD$ values if the data were normally distributed and as median (P25 ~ P75) values if the data were non-normally distributed. The chi-square test was used to compare categorical variables between MG-positive and MG-negative participants. Univariate analysis was used to evaluate factors associated with MG. A multivariate logistic regression model with stepwise variable selection was used to evaluate factors associated with MG based on the variables with a *P*-value of less than 0.1 in univariate analyses. For all analyses in this study, the results are reported as statistically significant when *P* ≤ 0.05. All data were analysed using SAS 9.4 (Statistics Analysis Systemint., Cary, NC, USA).

## Results

### Characteristics of the study participants

During the 17-month study period, 727 individuals were recruited, 49 of whom were excluded due to missing data. A total of 678 patients—489 (72%) men and 189 (28%) women—were finally included in the present study. The mean age of the study population was 34.02 ± 13.39 years (33.70 ± 9.23 years for the men and 34.83 ± 20.35 years for the women. Of the 489 men, 41 (6%) identified as gay and 8 (1%) as bisexual. None of the women identified themselves as lesbian or bisexual. Half of the participants were married, and 66% had completed higher education. Most (77%) had been living in Guangzhou for more than 6 months.

Among the 678 participants, 127 (14.2%) were symptomatic; 15.9% (108/678) of these were men, and 2.8% (19/678) were women. Other patient characteristics and MG test results are shown in Table 1.

**Table 1:** Demographic and behavioural characteristics of study participants attending the hospital STI clinic in Guangzhou, (*n* = 678)

Characteristics	Total	MG positive n (%) (percentage of individuals with the characteristic)	*P* value
Sex			0.827
Male	489	36 (7.36, 5.04–9.68)	
Female	189	13 (6.88, 3.24–10.52)	
Age (years)			0.158
18–25	136	15 (11.03, 5.70–16.36)	
26–45	474	30 (6.33, 4.13–8.53)	
> 45	68	4 (5.88, 0.15–11.62)	
Marital status			0.264
Single	309	23 (7.44, 4.50–10.39)	
Currently married	342	22 (6.43, 3.822–9.05)	
Other	27	4 (14.81, 0.49–29.14)	
Time at local residence			0.258
Less than 6 months	155	8 (5.16, 1.64–8.68)	
More than 6 months	523	41 (7.83, 5.53–10.15)	
Education level			0.329
Middle school or less	119	5 (4.20, 0.54–7.86)	
Senior school	109	8 (7.34, 2.37–12.31)	
Secondary school or junior college	203	13 (6.40, 3.00–9.80)	
Bachelor’s degree or higher	247	23 (9.31, 5.66–12.96)	
Sexual orientation			0.377
Heterosexual	629	47 (7.47, 5.41–9.53)	
MSM	49	2 (4.08, 0.00–9.82)	
Condom use in P6m			0.035
Always	76	1 (1.32, 0.00–3.94)	
Not always	602	48 (7.97, 5.80–10.14)	
Sexual partners in ^*^P6m			0.474
0–1	368	23 (6.25, 3.77–8.73)	
2 ~ 3	262	21 (8.02, 4.71–11.32)	
> 3	48	5 (10.42, 1.45–19.38)	
^**^Symptomatic			0.001
No	551	31 (5.63, 3.70–7.56)	
Yes	127	18 (14.17, 8.02–20.32)	

*P6m refers to the 6 months prior to interview; ** refers to the definition of “symptomatic” made in the measures part in the text

### MG prevalence among and clinical characteristics of the infected patients

MG was detected in 7.2% (49/678) of all patients in the study, corresponding to a prevalence of 7.4% (36/489) among men and to 6.9% (13/189) among women. Mg was more likely to be detected among symptomatic patients, the MG positivity rate was 14.2% among symptomatic patients, and 5.6% for asymptomatic patients, respectively.

Among patients with MG infection, only 36.7% (18/49) were symptomatic, of whom 50.0% (9/18) were co-infected with CT and/or NG. For detailed clinical manifestations, 16.7% male patients with Mg infection had abnormal physical examination findings, 47.2% had self-reported symptoms, 33.3% presented with excess leucocytes with microscopic examinations. For female patients with Mg infection, the rates for abnormal physical examination findings, self-reported symptoms and presented with excess leucocytes under microscopic examinations were 0%, 15,5, 53.8%, respectively. Clinical manifestations of Mg infected patients with or without other infections are shown in Table 2.

**Table 2:** Clinical manifestations of patients with MG who were coinfected with CT and/or NG,(*n* = 49)

		^a^MG (%)	^b^CT&MG (%)	^c^NG&MG (%)	^d^NG&CT&MG (%)	Total (%)
***Men (n = 36)***						
Physical examination findings	No	27 (90.0)	2 (6.7)	1 (5.3)	0 (0)	30 (83.3)
	Yes	3 (50)	0 (0)	2 (33.3)	1 (16.7)	6 (16.7)
Self-reported symptoms	No	17 (89.5)	1 (5.3)	1 (5.3)	0 (0)	19 (52.8)
	Yes	13 (76.5)	1 (5.9)	2 (11.8)	1 (5.9)	17 (47.2)
Presence of excess leucocytes	No	24 (100)	0 (0)	0 (0)	0 (0)	24 (66.7)
	Yes	6 (50)	2 (16.7)	3 (25)	1 (8.3)	12 (33.3)
***Women (n = 13)***						
Physical examination findings	No	10 (76.9)	3 (23.1)	0 (0)	0 (0)	13 (100)
	Yes	0 (0)	0 (0)	0 (0)	0 (0)	0 (0)
Self-reported symptoms	No	9 (81.8)	2 (18.2)	0 (0)	0 (0)	11 (84.6)
	Yes	1 (50)	1 (50)	0 (0)	0 (0)	2 (15.4)
Presence of excess leucocytes	No	6 (100)	0 (0)	0 (0)	0 (0)	6 (46.2)
	Yes	4 (57.1)	3 (42.9)	0 (0)	0 (0)	7 (53.8)

*a: MG infection; b:* Chlamydia trachomatis *infection; c: Neisseria gonorrhoeae infection; d:* Chlamydia trachomatis *and Neisseria gonorrhoeae infection.*

The data for co-infections in patients with MG infection are shown in Tables 2 and 3. According to our data, 10.2% of the patients were co-infected with CT (5/49), 6.1% with NG (3/49), 2.0% with both CT and NG (1/49), 8.2% with HSV-2 (4/49), 4.1% with TP (2/49) and 22.4% with GW (11/49).

**Table 3:** STI infections in patients with and without MG infection, (*n* = 678)

^a^STI	MG-negative	MG-positive	*P* value
^b^HIV			0.427
No	621 (92.7)	49 (7.3)	
Yes	8 (100.0)	0 (0.0)	
^c^CT			0.802
No	544 (92.7)	43 (7.3)	
Yes	85 (93.4)	*6 (6.6)	
^d^HSV-2			0.250
No	540 (92.3)	45 (7.7)	
Yes	88 (95.7)	4 (4.3)	
^e^TP			0.879
No	606 (92.8)	47 (7.2)	
Yes	23 (92.0)	2 (8.0)	
Genital warts			0.271
No	441 (92.1)	38 (7.9)	
Yes	188 (94.5)	11 (5.5)	
^f^NG			0.890
No	574 (92.7)	45 (7.3)	
Yes	55 (93.2)	*4 (6.8)	

^a^Sexually transmitted infection; ^b^ *Human immunodeficiency virus*; ^c^ Chlamydia trachomatis; ^d^ *Herpes simplex virus* 2; ^e^ Treponema pallidum; ^f^ *Neisseria gonorrhoeae*. * One patient had MG&CT&NG coinfection

### Infection rates for other STIs among the study subjects

The infection rates of the other STIs among the study subjects were as follows: CT, 13.4% (91/678); NG, 8.8% (59/678); HSV-2, 13.7% (92/678); GW, 29.4% (199/678); and HIV, 1.2% (8/678). Twenty-seven patients were co-infected with MG and one other STI. Sixteen patients were co-infected with MG and more than one other STI. The detailed data are shown in Table 3.

### Correlates of MG infections

Univariate logistic regression showed that symptomatic patients had a higher MG infection rate than asymptomatic patients (*P* = 0.001). Furthermore, patients who reported not using a condom during all sexual encounters were more likely to be infected with MG than patients who reported using a condom during each sexual encounter in the past 6 months (*P* = 0.035). Multivariable logistic regression showed that only clinical symptoms were associated with MG infection (Table 4). However, MG infection was not associated with other STIs, according to our analysis (Table 3).

**Table 4:** Regression analysis for factors associated with MG infection in Guangzhou, China, 2017–2018 (*n* = 678)

	MG			
	Univariate *OR*	*P* value	Multivariate *OR*	*P* value
Sex				
Male	Ref			
Female	0.93 (0.48–1.79)	0.827		
Age (years)				
18–25	1.98 (0.632–6.23)	0.241		
26–45	1.08 (0.37–3.17)	0.887		
> 45	Ref			
Marital status				
Single	Ref			
Currently married	0.855 (0.47–1.57)	0.612		
Other	2.16 (0.69–6.79)	0.186		
Time at local residence				
Less than 6 months	Ref			
More than 6 months	1.52 (0.73–3.17)	0.258		
Education level				
Middle school or less	Ref			
Senior school	1.81 (0.57–5.69)	0.313		
Secondary school or junior college	1.56 (0.54–4.49)	0.409		
Bachelor’s degree or higher	2.34 (0.87–6.32)	0.093		
Sexual orientation				
Heterosexual	1.90 (0.45–8.06)	0.377		
MSM	Ref			
Condom use in Past 6 m				
Always	Ref			
Not always	6.50 (1.11–8.66)	0.035		
Sexual partner in Past 6 m				
0–1	Ref			
2 ~ 3	1.31 (0.71–2.42)	0.393		
> 3	1.74 (0.63–4.83)	0.284		
Clinical symptoms				
No	Ref		Ref	
Yes	2.52 (2.03–3.13)	0.001	2.52 (2.03–3.13)	0.001

## Discussion

In our study, the prevalence of MG among patients attending our STI clinic was 7.2% (7.4% men, 6.9% women), and 36.7% of patients with MG infection were symptomatic. Among symptomatic patients, the prevalence of MG was 14.17% (18/127, 16 were men). Presentation with clinical symptoms was significantly associated with MG infection [OR = 2.52 (2.03–3.13)].

The strength of our study was that we tested a large number of STI attendees to provide data on the epidemiology of MG relative to other STIs, from a setting where MG testing is not routine. Thus, our data contributes to the sparse data on MG epidemiology, and could help in the development of national clinical guidelines for MG testing and screening protocols and the global efforts on MG monitoring. Limitations of our study included the inability to recruit larger numbers of female patients. Moreover, the patients were recruited from one hospital, and the results may thus not be generalizable to all patient populations in China.

The prevalence rates (7.2, 7.4%, respectively) among all patients and men attending our clinic were higher than those among patients attending STI clinics with symptoms of urogenital tract infections or for a check-up in Greece (5.7, 6.4%, respectively) [25] and Norway (4.9, 4.5%, respectively) [26] but lower than the corresponding rates in Denmark (9.0, 8.9%, respectively) [26], Sweden (9.8, 9.1%, respectively) [26] and the US (16.7, 17.2%, respectively) [27]. The MG prevalence among men with symptoms were 14.2% (18/127) in our study, 12.3% in a study in London [28] and 16.7% in a study in Brussels [29] The MG prevalence in women in our study was 6.9% (95% CI: 3.2–10.5%), similar to the rates in women attending STI clinics with symptoms of urogenital tract infections or for a check-up in Greece (6.9%) [25] and Norway (6.0%) [26] but lower than the corresponding rates in Denmark (9.3%) [26], Sweden (11.1%) [26] and in two studies in the US (16.3, 17.5%) [27, 30].

In our study, symptomatic patients were more likely to have MG infection than asymptomatic individuals (14.2% vs. 5.6%, *P* = 0.001). This pattern was also reported for men but not women among patients attending a young person’s clinic in Sweden [31]. However, some studies arrived at the opposite conclusions, i.e., finding no statistical association between clinical symptoms and MG infection in men attending an STI clinic in Guangxi Province, China [22], and no association between MG infection and microscopically defined urethritis or cervicitis in patients in Greece [25]. These different conclusions may be related to the composition of the sample and the definition of symptoms.

In studies from Greece [25], Kenya [32] and Honduras [33], consistent condom use was not protective against MG. In our study, in the univariate analysis, patients who reported not using a condom during all sexual encounters in the past 6 months were more likely to be infected with MG than patients who reported using condom during each sexual encounter in the past 6 months (*P* = 0.035), while the multivariate analysis result failed to verify the linkage between protection of Mg infection and condom use.

Consistent with previous findings from the UK [28] and Greece [34], no association of MG infection with other STIs was found in our study via the *chi-square* test, indicating that MG might be an independent pathogen in the genital tract. The high level of MG and CT/NG co-infection suggests that screening and treating CT may not greatly impact MG infection, since azithromycin (1 g), the first-line drug for treating CT infection, appears to be a suboptimal choice for MG treatment.

In conclusion, MG was one the most common pathogen causing infection in individuals attending an STI clinic in Guangdong (7.2%). MG was an independent infection, and symptomatic patients were more likely than asymptomatic patients to be infected. Improving the accessibility of Mg testing in STI clinics in Guangdong Province is necessary, and more epidemiological studies are needed to provide evidence for future testing guidelines.

## Data Availability

The data sets generated and/or analysed during the current study are available from the corresponding author upon reasonable request.

## Abbreviations

MG: *Mycoplasma genitalium*
STI: Sexually transmitted infection
GW: Genital warts
HSV: Herpes simplex virus
NG: *Neisseria gonorrhoeae*
CT: *Chlamydia trachomatis*
